# Maculopathy in Dengue Fever

**DOI:** 10.3201/eid1204.051476

**Published:** 2006-04

**Authors:** Daniel Hsien-Wen Su, Soon-Phaik Chee

**Affiliations:** * Singapore National Eye Centre, Singapore;; † National University of Singapore, Singapore;; ‡ Singapore Eye Research Institute, Singapore

**Keywords:** dengue fever, dengue maculopathy, retinal hemorrhages, metamorphopsia, blurring of vision, letter

**To the Editor:** A recent article by Chlebicki et al (*1*) described 4 patients hospitalized for dengue fever who were found to have retinal hemorrhages. These patients reported reduced visual acuity and metamorphopsia, i.e., distorted visual images attributable to intrinsic retinal disease involving the macula; macular hemorrhages and exudates were found on retinal examination. The authors concluded that the retinal hemorrhages were responsible for the patients' visual symptoms.

This conclusion is misleading because retinal hemorrhages alone cause scotomas. Rather, the accumulation of subretinal fluid in the macula results in metamorphopsia and blurring of vision. In previous reports of patients in whom macular changes developed from dengue fever, some were found to have macular hemorrhages (*2**–**4*). In addition, clinical examination and investigation of these patients showed vasculopathologic changes in the macular region that affected the retinal and choroidal blood vessels (*5*), although the tissues of the periphery tended to be spared. A fluorescein angiograph of the retina showed knobby hyperfluorescence of the retinal arterioles with minimal leakage, as well as some spots of leakage at the level of the retinal pigment epithelium. An indocyanine green angiograph showed diffuse hyperfluorescence of the choroid. These pathologic changes in the macula were the most likely cause of the blurring of vision in such patients, which has been the case in our experience.

The article by Chlebicki et al. did not state whether these procedures had been performed on their patients to confirm or exclude retinal or choroidal vasculopathy in the macula. Therefore, these authors would have had difficulty concluding that retinal hemorrhages caused blurring of vision and metamorphopsia in patients with dengue maculopathy.

## References

[1] Chlebicki MP, Ang B, Barkham T, Laude A. Retinal hemorrhages in 4 patients with dengue fever. Emerg Infect Dis. 2005;11:770–2. 15890137 10.3201/eid1105.040992 PMC3320366

[2] Wen KH, Sheu MM, Chung CB, Wang HZ, Chen CW. The ocular fundus findings in dengue fever [article in Chinese]. Gaoxiong Yi Xue Ke Xue Za Zhi. 1989;5:24–30. 2733064

[3] Haritoglou C, Scholz F, Bialaslewicz A, Klauss V. Ocular manifestations in dengue fever [article in German]. Ophthalmologe. 2000;97:433–6. 10.1007/s003470070094 10916388

[4] Haritoglou C, Dotse SD, Rudolph G, Stephan CM, Thurau SR, Klauss V. A tourist with dengue fever and visual loss. Lancet. 2002;360:1070. 10.1016/S0140-6736(02)11145-7 12383989

[5] Lim WK, Mathur R, Koh A, Yeoh R, Chee SP. Ocular manifestations of dengue fever. Ophthalmology. 2004;111:2057–64. 10.1016/j.ophtha.2004.03.038 15522372

